# Neuroimaging of HIV-associated neurocognitive disorders

**DOI:** 10.1590/1980-57642015DN94000380

**Published:** 2015

**Authors:** Michel Elyas Jung Haziot, Silas Pereira Barbosa Junior, José E. Vidal, Francisco Tomaz Meneses de Oliveira, Augusto César Penalva de Oliveira

**Affiliations:** 1 Neuroscience Research Group / Institute of Infectious Diseases Emílio Ribas of the University of São Paulo, SP, Brazil.; 2 Neurology Department of the Santa Casa de São Paulo, SP, Brazil.; 3 Department of Infectious Diseases of the University of São Paulo, SP, Brazil.

**Keywords:** HIV, neuroimaging, HAND, dementia, HIV, neuroimagem, HAND, demência

## Abstract

A significant increase in the incidence of cognitive impairment in HIV/AIDS patients has been continuously observed. Consequently, three classification categories of cognitive impairment have been proposed: asymptomatic neurocognitive impairment (ANI) and mild neurocognitive disorder (MND), that correspond to the mild and intermediate forms, and HIV-associated dementia (HAD) for the most severe cases. HIV-associated neurocognitive disorders (HAND) is a broad term that encompasses these three categories. Moreover, the application of neuroimaging methods has led to a major breakthrough in understanding of the neurological changes in HIV, providing greater reliability in the exclusion of associated diseases and allowing earlier diagnosis. Therefore, abnormalities and/or specific neuroimaging elements may soon be incorporated into the HAND classification criteria, which will be of great value in the management of these diseases, including in the optimization of high CNS penetration antiretroviral regimens.

## Abstract (pt)

Um aumento expressivo da incidência de alterações cognitivas em pacientes com AIDS/HIV vem ocorrendo, assim três categorias de acometimento cognitivo foram propostas: comprometimento cognitivo assintomático (ANI) e alteração cognitiva leve/moderada (MND) que correspondem às formas leves e intermediárias; e demência associada ao HIV (HAD), a forma mais grave. O termo HIV-associated neurocognitive disorders (HAND) é uma denominação genérica que engloba as três categorias de acometimento. A aplicação de métodos de neuroimagem agregou grande avanço no entendimento das alterações neurológicas do HIV, promovendo maior confiabilidade na exclusão de patologias associadas e diagnóstico mais precoce. Diante disso, as alterações ou características específicas de neuroimagem podem ser em breve incorporadas aos critérios de classificação da HAND, o que será de grande valor no seu manejo, inclusive no auxílio para a otimização de esquemas antirretrovirais de alta penetração no SNC.

## INTRODUCTION

The introduction of antiretroviral combination therapy in the 1990s led to a dramatic change in the natural history of infection by the human immunodeficiency virus type-1 (HIV-1).^1^ Previously, AIDS was a rapidly progressive and fatal condition whereas now it can be considered a chronic manageable disease. With over 40 million people infected worldwide, HIV currently has a high prevalence of cognitive impairment, even in individuals in regular use of highly active antiretroviral combination therapy (HAART).^2^ [The association between HIV-1 infection and dementia is not a new concept, where HIV-1 neurotropism has been known since the beginning of AIDS epidemic.^3^ Navia et al. in 1986^4^ first described the clinical and neuropathological findings in patients at terminal stages of the disease presenting cognitive decline, impaired motor function and behavioral changes, called AIDS dementia complex (ADC) or HIV-associated dementia (HAD) - a severe form CNS involvement by the HIV-1.

The introduction of HAART has decreased the incidence of most opportunistic diseases, and had a highly positive effect on the morbidity and mortality of HIV-1 infected individuals. Concomitantly, the incidence of severe cognitive disorders sharply decreased among these patients in the past 15 years. Although the incidence of HAD has declined, a paradoxical apparent increase in the incidence of neurocognitive dysfunction^5^ has been observed. One possible explanation for this phenomenon is the more prolonged direct neurotropic effect of HIV-1 in populations that now have longer survival.^5^

It is believed that 50% of patients with HIV-1 present some degree of cognitive dysfunction attributed directly to the virus.^6^ The epidemiological evolution of this process was a reduction in severe forms (HAD) and an increase in mild and intermediate cognitive dysfunction cases. Currently, prevalence rates are estimated to be 15-30% for mild /subclinical forms, 20-50% for moderate forms, and 2-8% for severe forms.^7,8^

The immune scenario has also become more intricate, since all categories of HAND can be observed in individuals with moderate or even very mild degrees of immunosuppression. The diagnosis of such a spectrum of cognitive changes is complex as it involves excluding other associated diseases, differential diagnosis with other dementias unrelated to HIV, application of formal neuropsychological evaluation, and the absence of a specific biomarker to date. In this context, neuroimaging methods are gaining increasing prominence; conventional MRI techniques have improved and new built-in analysis methods have been developed, including some already available for clinical use. Neuroimaging is primarily employed to exclude HAND mimicking causes, but its usage can be extended to the diagnosis, prognosis and evaluation of therapeutic response in patients with HAND.^2,9^

## CLASSIFICATION AND DIAGNOSIS

With the increase in less severe forms of cognitive impairment, a new classification was proposed in 2007 (also known as the Frascati criteria), based on the degree of impairment in performing activities of daily living and performance on neuropsychological tests. Cognitive impairment is classified into three categories: asymptomatic neurocognitive impairment (ANI) and mild neurocognitive disorder (MND), that correspond to the mild and intermediate forms; and HIV-associated dementia (HAD), the most severe form (Table 1). HIV-associated neurocognitive disorders (HAND) is a broad term that encompasses these three categories.^10^ New neuroimaging methods parameters may soon be incorporated into these criteria as they represent a non-invasive way of measuring the degree of brain damage caused by HIV-1 in this population.^11^

**Table 1:** Classification of clinical forms of HIV neurocognitive impairment.

ANI - asymptomatic neurocognitive impairment	MND - mild neurocognitive disorder	HAD - HIV-associated dementia
Changes in ≥ 2 cognitive domains on neuropsycho­logical assessment, but without functional impair­ment in activities of daily living.	Changes in ≥ 2 cognitive domains on neu­ropsychological assessment, with mild to moderate functional impairment, in activities of daily living.	There are serious changes in ≥ 2 cognitive domains, with severe impairment in activi­ties of daily living.

The diagnosis of HAND, suspected in the initial clinical evaluation (impaired attention, executive and memory functions), is confirmed by neuropsychological tests, where cognitive impairment must be exclusively attributed to HIV-1 action, after excluding other causes that may justify or mimic the disease. Although there are no confirmatory or highly specific radiological findings for HAND, the importance of neuroimaging goes beyond the differential diagnosis of infectious or metabolic processes that mimic HIV-1 cognitive primary changes. Neuroimaging methods can also be useful in the diagnosis of other dementias unrelated to HIV and for the evaluation of response to treatment.^12^ The main risk factors associated with HAND should be considered:

1) nadir CD4 cell count < 200 cells / uL;
2) age> 50 years;
3) hepatitis C virus (HCV) coinfection;
4) diabetes or insulin resistance; and
5) cardiovascular disease.^8,13^

## NEUROIMAGING METHODS

**Conventional Computed Tomography (CT) and Magnetic Resonance Imaging (MRI).** An anatomical imaging study with CT or MRI is often the initial step in the diagnostic approach for HAND. As mentioned previously, although there are no specific radiological findings capable of confirming the diagnosis of HAND, a neuroimaging study is initially required to exclude diseases that can mimic HAND. Moreover, it is highly unlikely that no suggestive changes on MRI examinations are evident in cases of serious neurological impairment, such as HAD; similarly, an increasing number of radiological findings associated with mild and intermediate forms (currently the most prevalent) are also being observed.^16^

The most common abnormalities, while considering the limits and difference in methods, observed on conventional CT/MRI associated with HAND are:

(i) gray matter atrophy of the cerebral cortex, particularly the anterior cingulate gyrus, lateral temporal cortex, primary sensory and motor cortex areas, and parietal and frontal lobes;
(ii) MRI signal intensity changes and atrophy of the deep white matter; some patients present focal subcortical white matter involvement that can resemble the abnormalities seen in progressive multifocal leukoencephalopathy (PML);
(iii) volumetric reduction of the basal ganglia associated with motor impairment, in addition to the cognitive symptoms;
(iv) loss of white matter integrity in the corpus callosum and corona radiata;
(v) at later stages, confluent and bilateral symmetrical lesions in white matter, predominantly affecting the periventricular regions and semioval centers with relative sparing of the subcortical white matter and posterior fossa.

**Figure 1:**
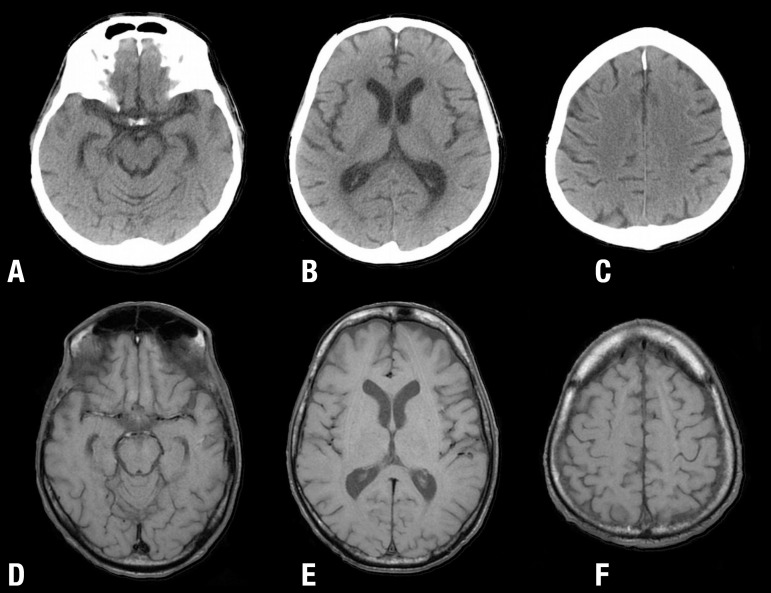
[A, B, C] Conventional CT and [D, E, F] MRI T1W images of two different patients with HAND, showing changes of cerebral atrophy with enlargement of most CFS-containing spaces, including basal cisterns, Sylvian fissures, cerebral ventricles and cortical sulci.

**Figure 2:**
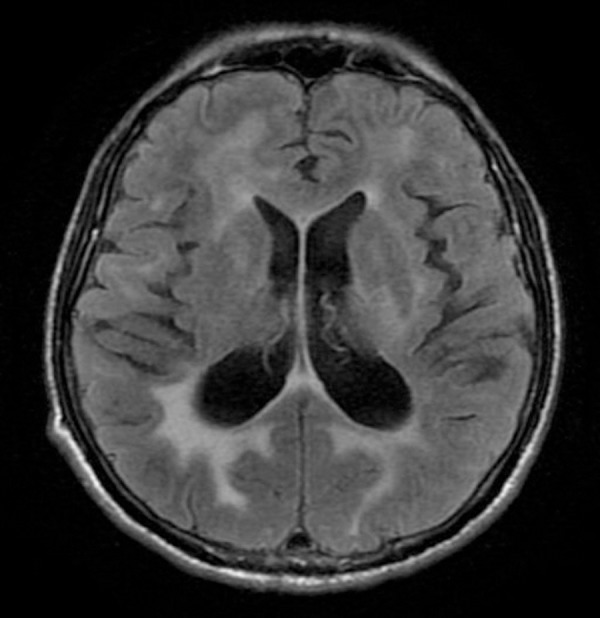
MRI FLAIR image of patient with HAD, depicting high signal intensity in the periventricular and deep white matter. Note the relatively symmetrical involvement of the white matter, uncommon in other viral demyelinating diseases such as Progressive Multifocal Leukoencephalopathy (PML).

**Figure 3:**
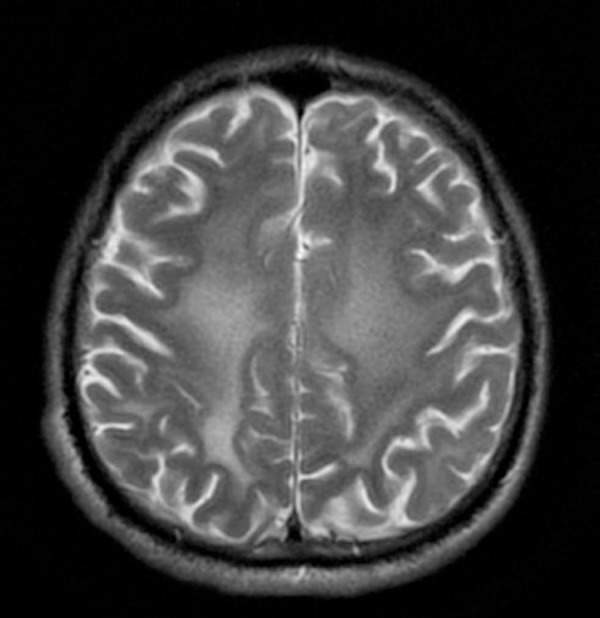
MRI T2W image of patient with widespread leukoencephalopathy involving the centrum semiovale.

While these findings are more suggestive and more frequently seen, it is not uncommon to observe an unequal distribution of lesions in the white matter or a diffuse pattern, associated with prominent cortical sulci and widened lateral ventricles^17^ (Figures 1, 2 and 3).

In an attempt to predict a more aggressive or milder evolution of neurocognitive disorders associated with HIV-1,^7,10,12,15^ several studies have been conducted to show a correlation between imaging findings *in vivo* with those detected after death in patients infected with HIV. A study conducted at a San Diego-US Psychiatry Department^7^ demonstrated a great number of HIV pathological findings and dendritic loss on autopsy of HIV-infected patients without other brain infections. These results were associated with signal abnormalities in white matter of the brain on MRI shortly before their death, and leukoencephalopathy was correlated with diffuse white matter hyperintensity on MRI images.^7^

Volumetric structural neuroimaging of regions of interest in the brain of individuals with HAND have been compared with non-HIV healthy controls. A common observation in the pre-HAART era was of subjects with HAD presenting volumetric loss in the basal ganglia, posterior cortical region and white matter. These findings sparked the use of more sophisticated methods of measuring the volume of these areas in patients with less severe forms of cognitive dysfunction.^18^ Conventional MRI devices with magnetic fields of between 1.5T and 3T are employed to perform volumetric studies using gradient echo (MPRAGE) sequences that generate high-resolution T1-weighted images. These images provide better differentiation between gray/white matter and CSF, allowing more accurate volume measurements.^20^ Currently, HIV-1 patients on HAART therapy, with or without HAND, present both subcortical and cortical atrophy, suggesting loss of brain volume despite the use of effective treatment. It is well established that individuals with worse neurocognitive performance have smaller brain volumes. New studies seek to correlate not only neurocognitive performance with volumes, but also specific virological markers and the HIV-1 viral load in the CSF. A major current limitation in the evaluation of brain atrophy is the volume reduction that occurs naturally in older individuals, an age group that represents a large proportion of the HIV-1 infected population, a factor which could lead to overestimation of the degree of volumetric loss that is due to the direct action of the HIV-1.^21^

## Functional and experimental methods

*Proton magnetic resonance spectroscopy (MRS)* is a functional MRI imaging technique that measures brain metabolites and is the most frequently used method for the study of HAND.^18^ The molecules typically measured in the diagnosis of HAND are: N-acetyl-aspartate (NAA), a neuronal marker; choline (Cho), a marker of cell proliferation and inflammatory response; creatine (Cr), an indirect measure of brain metabolism and reference marker; and myo-inositol (mI), a tissue glial marker. Increases in Cho and mI are observed in almost all cases of HIV infection, even in early stages or totally asymptomatic individuals. However, NAA and its NAA/Cr ratio are normal or altered depending on the degree of cognitive dysfunction.^19^ MRS can be very useful in the assessment and follow-up of patients with HAND, particularly to determine their therapeutic response. Although there are several inherent limitations, MRS is more sensitive than conventional MRI alone in the detection and monitoring of patients with HAND. The implementation of routine MRS in neuroimaging HAND protocols can provide more efficient diagnosis and monitoring of patients, although additional longitudinal studies are still needed in this regard.^20^

*Diffusion Tensor Imaging (DTI)* is a powerful technique for microstructural evaluation of brain tissue, and its use in HAND is focused on the assessment of the structural integrity of white matter.^18^ DTI measures the diffusion of water molecules through brain tissue. Unlike the conventional diffusion technique (DWI), DTI can disclose those white matter tracts, or tractography, which seem to be damaged in patients with HAND and correlates both with clinical and evolution findings. Conflicting results were observed in the few studies that have investigated the impact of DTI in HAND, but DTI appears to be a promising method.^13,22^

*Functional Magnetic Resonance Imaging (fMRI)* is a neuroimaging method for evaluating metabolic activity in areas of interest of the brain, in contrast to other methods that evaluate the integrity of the structures of these areas. There are several methods for acquiring fMRI images, with most HAND studies investigating the utility of the blood oxygen level dependent (BOLD-fMRI)^18^ technique. Basically, FMRI-BOLD measures the degree of coupling between neuronal activity in a region where aa specific task is to be carried out, and blood flow to that area - and thus can infer whether a given region is more or less active when a particular stimulus or task is performed by the patient.^23^ HIV-positive patients have high activity in the lower region of the left frontal gyrus and caudate nucleus compared with HIV-negative individuals. The degree of decoupling of this frontal striatal network correlates with the degree of cognitive impairment. Another distinguishing feature is the reduction of all measurable BOLD-fMRI neural networks in individuals with HAND compared with those with other neurodegenerative dementias [18]. fMRI has been studied in small groups of individuals with HAND, and longitudinal studies with larger groups are needed to validate the technique for clinical use.^24^

*Positron Emission Tomography (PET)* Fluorodeoxyglucose (FDG)-PET is a commonly used method in clinical practice to indirectly measure the metabolic cell/tissue activity through the use of fluorodeoxyglucose. This technique has not yet shown immediate clinical applicability in managing HAND. However, it has cast light on several pathophysiological concepts that remain unclear. This technique demonstrated that metabolic changes in brain tissue secondary to HIV-1 occur long before any measurable structural change can be detected. Other issues still under study with PET are dopaminergic system metabolic activity interaction, the degree of cognitive impairment, and the role that specific neurotransmitters such as serotonin play in pathophysiological processes.^18,20^

## THE ROLE OF NEUROIMAGING LOOKING FORWARD

The application of neuroimaging methods, particularly MRI, marked a major advance in understanding of neurological changes in HIV-1 infected patients. In face of the need for a more accurate and earlier diagnosis, abnormalities or specific neuroimaging features may in the future be incorporated into the HAND classification criteria.^25,26^ Despite the advancement of these complementary investigation methods, there is still much to learn about their application and relevance in patients with HAND. Most studies have compared HIV-positive individuals with HIV-negative controls, focusing on the diagnosis of HAND or exclusion of associated comorbidities.^21^ Further studies are needed investigating the pathophysiology of the disease in the whole brain. These studies may help to predict which HIV-infected patients are at increased risk for HAND, and thus improve the management of the disease, such as by optimization of high CNS penetration antiretroviral regimens capable of blocking viral exposure, which could be incorporated into AIDS therapeutic guidelines.^13,27,28^
